# Evalutation of hand hygiene compliance in medical educational videos

**DOI:** 10.1186/1753-6561-5-S6-O70

**Published:** 2011-06-29

**Authors:** Y Longtin, J Longtin, H Sax, D Pittet

**Affiliations:** 1 Infectious diseases, Centre Hospitalier Universitaire de Québec, Québec, Canada; 2 Infection Control Service, Geneva University Hospitals, Geneva, Switzerland

## Introduction / objectives

Hand hygiene (HH) is widely regarded as the single most effective measure to prevent healthcare-associated infections. However, physicians’ adherence to HH remains poor. Lack of knowledge regarding the proper indications is a known factor contributing to low compliance. However, teachings regarding this basic safety procedure are often overlooked when designing teaching material.

## Methods

Observational study of clinical videos published in *the New England Journal of Medicine* between Apr. 2006 and Dec. 2010 to measure reference to HH as recommended by WHO guidelines. An opportunity was defined as a need to perform HH directly related to the procedure.

## Results

29 videos (total running time, 3h42min) depicting 17 sterile, 11 aseptic, and 5 clinical procedures were reviewed. Sixty-six opportunities to explicitly mention the need to perform HH were recorded. Indication to perform HH was properly mentioned 24% (16/66) of time. Proper reference to HH was higher before aseptic procedures (9/14 opportunities [64%]) and before performing a sterile task (7/16 opportunities [43%]). However, explicit indication to perform HH before touching a patient, after body fluid exposure and after touching a patient were never mentioned. Hand washing was more frequently mentioned than hand rubbing with an alcohol-based solution (ratio, 6:1). Additional information regarding HH was present in 3/29 (10%) of written supplements. Respect of guidelines regarding jewellery was high (28/29 videos). Gloves were uniformly used whenever indicated. However, 10 of the 29 videos (34%) depicted ≥1 sequence in which gloves were used despite a clear need to do so.

## Conclusion

There is a need to adequately portray proper hand hygiene and glove use in teaching material aimed at physicians.

## Disclosure of interest

None declared.

